# Abstract of Proceedings of the Aurora City Medical Association

**Published:** 1858-11

**Authors:** D. W. Young

**Affiliations:** Secretary


### ABSTRACT OF PROCEEDINGS OF THE AURORA CITY MEDICAL ASSOCIATION.

*Monday Evening*, *Aug.* 2*nd*, 1858.

The Association met at Dr. Allaire's office.

*Present.*—The President, Dr. P. A. Allaire, in the chair; Drs. Winslow, Hard, Gillett and Young.

Minutes of the last meeting read and approved.

Dr. Hard reported the following case of "*Uterine Abscess opening into the Rectum:*"

*May* 30*th*, 1858.—I was called in haste to see Mrs. E., a married lady, aged twenty, of good constitution, and the mother of two healthy children. A few months previous to this time, she aborted, having been pregnant about two and a half months, from which she recovered without anything occurring worth recording. She again became pregnant, and had arrived at about the same stage of utero-gestation; and while engaged in cleaning house (washing windows), she was taken with labor pains and hemorrhage so severe that she could not leave the room, or even get upon a bed, but sank down upon the floor and called for help. In a few minutes the fœtus and placenta were expelled entire. Some women who came in, laid her on the bed and sent for me. I arrived about half an hour after the expulsion of the fœtus and placenta. Her lips were pale and almost bloodless, she had vomited freely. Skin cold and clammy. Pulse scarcely perceptible at the wrist, and she was so nearly comatose and insensible that it was with much difficulty she could be aroused to consciousness. I immediately applied my left hand to the abdomen, and with my right made examination per vaginum. I found the hemorrhage very profuse and the aorta pulsating rapidly; I compressed the aorta with the left hand to control the hemorrhage if possible until other means could be resorted to, and also applied cloths wet in cold water over the uterine region and introduced others into the vagina (there being no ice at hand), and ordered attendants to administer pulv. ergot., tinct. capsicum and carb. ammonia, which were freely given. In this critical state I did not stop to measure doses very precisely. We also applied warm bricks and sinapisms to the extremities. The hemorrhage was soon arrested, and she gradually aroused from the stupor. From the great loss of blood and the impression made upon the nervous system she did not soon recover. The pulse became frequent and small, with considerable febrile excitement. I continued to visit her till June 19, she then being able to sit up for an hour at a time. After this she continued to improve so that she rode out and also walked to the neighbors, some forty rods distance.

*July* 1*st.*—I was summoned in the night to see her again. I found her in great pain. The abdomen distended, tympanitic, and so sensitive to the touch that she could not bear the weight of the bedclothes. The greatest tenderness was found in the iliac and hypogastric regions. The expression of the countenance was anxious. Pulse small and 135 beats to the minute. Bowels had not moved for two or three days. I learned that two days before this she had visited a sick neighbor, and had also remained a few hours in a room while being whitewashed. My diagnosis was metritic peritonitis.

Having been greatly reduced by hemorrhage a few weeks before, I did not resort to venesection. I gave her a cathartic of calomel, to be followed by sulph. magnesia. Trusted to "*tinct. veratrum viride*" to control the pulse, used opium to allay pain and nervous irritability, and applied fomentations to the abdomen. After a movement of the bowels was obtained, to secure which we had to resort to enemata, I gave calomel in small doses, and followed the course usually recommended by standard authors, with the addition of the tinct. verat. viride, which controled the action of the heart most admirably, and to which I would particularly call the attention of this society.

The peritonitis gradually subsided. After about two weeks, by examinations per vaginum and rectum, I became satisfied there must be an abscess forming in the posterior part of the uterus. The os uteri appeared natural, except being nearer the pubis, and no part that could be reached by the finger appeared tender but that posterior to the os uteri, which point, the moment it was touched by the finger, gave great pain. The same occurred when the examination was made by the rectum. Pressure of the hand upon the bowels above the pubis produced pain in the pelvis, which extended to the iliac regions, more severe in the left than the right. Every evacuation of the bowels gave excruciating pain so severe that the thought that she must soon have a movement of the bowels, would throw her into a profuse perspiration, and so agitate her, that an anodyne was required to restore quiet. The treatment consisted of counter-irritants, anodynes and alteratives, and in the later stage, tonics and nutritious diet, to sustain the rapidly failing powers.

*July 19th.*—I was summoned and found my patient greatly prostrated. Pulse small and feeble. The abscess had just discharged into the rectum, and she had passed about half a pint of well formed pus. I immediately gave wine and tonics. The discharge of pus continued for a week, when it ceased, and she has improved ever since. This is a brief account of the case, with the treatment. All the minutiæ I have not thought best to report; for I have presented this case not so much on account of what has occurred (although I do not recollect of ever hearing or reading of a case of uterine abscess which discharged into the rectum), as to propose a question for the consideration of this society which seems to me to be of the greatest importance. What would be the result should she again become pregnant? Adhesions must have formed between the uterus and the rectum. Could the uterus rise from the pelvis as gestation advanced? Would another abortion take place, or would it be likely to compromise the life of the mother?

Dr. Winslow thought that it would be likely to compromise the life of the mother if she should again become pregnant, as he thought that it would be impossible for the uterus to rise from the pelvis if there were adhesions between it and the rectum.

Dr. Gillett was of opinion that if there were adhesions formed to any great extent, successful parturition could hardly be anticipated.

Dr. Allaire thought that she would be very likely to abort again if she should become pregnant very soon—he thought that she ought to be advised accordingly.

Dr. Hard thought it very likely to compromise the life of the woman, should she become pregnant again, and said he should advise her and her husband of the probable consequences of another pregnancy.

Dr. Young thought *that if there really was adhesion* between the rectum and uterus, abortion would be more likely to take place at an early stage of pregnancy than before the adhesions.

Dr. Winslow reported a case of monstrosity.

He was called a few weeks since to visit a patient in labor with her first child. The labor was natural but rather slow, and when the child was born it was found to have a tumor five or six inches long, and (I think) about two to four in circumference, and covered with hair for about half its extent. The tumor was smallest at its attachment to the scalp. The bones of the head were not very well developed. Dr. Allaire was called and removed the tumor. The wound healed kindly and the child done well. The monstrosity seemed to be the result of excessive development.

The mother thought that it was caused by a fright. Sometime during the first months of her pregnancy, her husband, who had just killed a hog, came into the house, and unbeknown to her slipped a kidney down her back inside her clothing, which frightened her very much. She attributes the monstrosity to the kidney. The tumor, however, did not resemble a kidney in form or appearance.

On motion the association adjourned to meet at Dr. Allaire's office, on the first Monday evening in September.

D. W. YOUNG, M.D., *Secretary*.

